# Investigation of the Microstructures and Mechanical Properties of Sn-Cu-Bi-In-Ni Solders

**DOI:** 10.3390/ma18040858

**Published:** 2025-02-16

**Authors:** Xiaochun Lv, Chenghao Zhang, Yang Liu, Zhen Pan, Zhiyuan Wang, Fenglian Sun

**Affiliations:** School of Materials Science and Chemical Engineering, Harbin University of Science and Technology, Harbin 150080, China; lvxiaochun@163.com (X.L.); yang_liu@hrbust.edu.cn (Y.L.); panzhen@hrbust.edu.cn (Z.P.); wangzhiyuan@hrbust.edu.cn (Z.W.)

**Keywords:** Sn-Cu-Bi-In-Ni solder, (Cu,Ni)_6_Sn_5_, shear strength, vibration lifetime

## Abstract

The development of Ag-free Sn solders has attracted significant attention due to the requirement of high-density electronic packaging. In this study, we investigate the Ni element on the microstructures and mechanical properties of Ag-free Sn-Cu-Bi-In solders. This paper details the microstructures and phases of the as-prepared Sn-Cu-Bi-In-Ni solders, as well as its mechanical properties. Specifically, the intermetallic compound (IMC) Cu_6_Sn_5_ is observed to be distributed in the Sn matrix, forming near-eutectic structures. The incorporation of Ni into Sn-Cu-Bi-In enhances the mechanical properties of the solder joints, including the shear strength and vibrational stability. In the joint obtained using the as-prepared Sn-Cu-Bi-In-Ni solders, a (Cu,Ni)_6_Sn_5_ IMC layer forms at the interface between Sn ball and Cu pad. The beneficial effects of Ni can be primarily attributed to its ability to adjust the mechanical properties and thermal expansion, enhancing the stability of solder joints. A TEM analysis reveals the closely packed atomic interface of Cu/(Cu,Ni)_6_Sn_5_ and (Cu,Ni)_6_Sn_5_/Sn, elucidating the joining mechanism involved.

## 1. Introduction

The advancement in electronic packaging technology has led to a growing demand for high-density solder joints, particularly in sectors such as automotive electronics, medical devices, and display device [1,2]. To address the complexities of various application scenarios, these high-density solder joints must demonstrate superior mechanical properties, encompassing both static and dynamic loads, thereby ensuring long-term reliability [3,4]. Traditionally, Ag-containing solders, such as Sn96.5Ag3Cu0.5 (SAC305) [5], Sn98.5Ag1Cu0.5 (SAC105) [6], and Sn3.5Ag1.5Bi [7], have been utilized for their excellent mechanical characteristics. However, Ag-based solders are prone to electrochemical migration issues, which can compromise the functionality of high-density solder joints [8,9]. Consequently, there is an urgent need to develop high-performance Ag-free Sn-based solders that cater to the demands of contemporary electronic packaging.

Developing such advanced Ag-free Sn-based solder materials involves addressing key challenges. In the Sn-Cu system, the incorporation of Ag often results in inferior mechanical properties when compared to traditional Ag-containing solders used in electronic packaging [10]. A Sn-Au alloy system could be applied to a variety of substrate materials and possess high mechanical strength and thermal properties [11]. In practical applications, such as BGA packaging, the common Sn-based solders without a noble metal attract more attention due to the cost issues. To enhance the thermal and mechanical properties, two primary types of elements are commonly added to the solder alloy: low-melting-point components and high-melting-point components. The former aids in reducing the melting temperature of the solder, thereby minimizing thermal impact during the reflow process and improving wettability. Common examples of low-melting-point components include In, Sb, Bi, and Zn [12,13,14,15]. These elements can form eutectic alloys with Sn, significantly lowering the melting point of the solder alloy while maintaining excellent mechanical properties and reliability. Zeng et al. [16] conducted an investigation into the microstructures of Sn-Cu-Zn solders, demonstrating that Zn is uniformly distributed within the eutectic structure. The CuZn compound was formed during solidification. Tian [17] investigated that Sn-0.7Cu solders exhibit a lower melting temperature upon the addition of In. The presence of the Cu_6_(Sn,In)_5_ compound in the Sn-Cu-In solders contributes to solution strengthening and stress relaxation.

On the other hand, components with high melting points, such as Ni [18], Cr [19], and various rare earth elements [20,21], can also be incorporated to enhance the thermal and mechanical properties of Sn-based solders. The addition of these trace elements to Sn-based solder results in lattice distortion, thereby improving the wettability and strength, and significantly enhancing the overall performance of the solders. The inclusion of high-melting-temperature compositions can optimize the microstructure of Sn-Cu-based solders by reducing Sn grain sizes, adjusting the morphologies and doping of Cu-Sn compounds, and generating new types of compounds [22]. Sn-Cu-La has been proven to affect the growth of Cu_6_Sn_5_ IMC, promoting a uniform distribution of IMC through the adsorption effect [23]. Furthermore, La can increase the nucleation rate by lowering the nucleation energy. Bang et al. [24] discovered that adding Cr to the Sn/Cu system results in the formation of CrSn_2_ at the grain boundary of Cu_6_Sn_5_, which increases the activation energy and inhibits the growth of IMC layer. Ni is one of the most extensively researched compositions in the Sn-Cu system [25]. An addition of 0.05 wt.% Ni element can improve the wetting behavior of Sn-Cu solders, and the microstructures of the Sn-matrix can be significantly refined by providing the nucleation sites. The effect of Ni on the Cu_6_Sn_5_ compound includes phase stabilizing, mechanical properties, and reduction in IMC thickness [26,27].

For the mechanical stability of solder joints, especially under dynamic load, the solder joints should exhibit good strength and plasticity, as well as the ability to relax residual stress [28]. To achieve these requirements, a novel combination of low- and high-melting-temperature compositions could be an optimal strategy. The elements of In, Bi, and Ni was introduced to the Sn-Cu system in this research. Compared to previous study, the synergetic effect of these elements could guarantee the thermal and mechanical properties of Ag-free solders.

In this study, Sn-Cu-Bi-In-Ni solders were first prepared by vacuum smelting. The microstructures and phase composition of the prepared solders were characterized and discussed. After reflow soldering, the interfacial structures and shear strength of the solder joints were investigated. The atomic interface of different phases in the solder joint were logically analyzed. The vibration stability was evaluated by observing the interfacial cracks and comparing the lifetime.

## 2. Experimental Section

### 2.1. Vacuum Smelting of Sn-Cu-Bi-In-Ni Alloys

**Figure 1:**
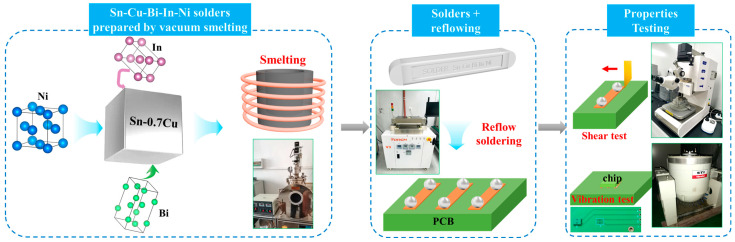
Schematic diagram of preparing Sn-Cu-Bi-In-Ni solder alloys, reflow soldering, shear test, and vibration test.

The experimental procedure conducted in this study is illustrated in Figure 1. Sn-Cu-Bi-In-Ni alloys were prepared using vacuum smelting of high-purity (99.99%) Sn, Cu, Bi, In, and Ni particles, which were purchased from Beijing Tim New Materials company (Beijing, China). The weight percentages of Cu, Bi, In, and Ni is 0.7%, 1%, 3%, and 0.05%, as shown in Table 1. The 0.7% percentage of Cu is a commonly used content for Sn-Cu-based solders. These amounts of Bi and In could lead to good thermal and mechanical properties, as shown in our previous study [29]. The percentage of Ni element was chosen based on the current research [25] and our preliminary experiments. The smelting process was carried out in a graphite crucible in a vacuum melting furnace. The melting temperature was set at 550 °C, with a holding time of 50 min, ensuring the sufficient melting and reaction.

### 2.2. Reflow Soldering

The solder balls were achieved by cutting the as-prepared alloys into small pieces and subsequently melting them in the glycerol. The glycerol acts as an efficient heat transfer medium and creates a protective environment. In addition, the surface tension of glycerol helps the formation of spherical solder balls. After cooling, the solder balls were ultrasonic cleaned in ethanol for 15 min.

The sizes of the chip and PCB board are 7.8 × 6.2 × 1 mm and 132 × 77 × 1 mm, respectively. The diameter of the Cu pads is 300 μm, and the diameter of the ball is 500 μm. The reflow soldering process was carried out by placing the solder balls on Cu pads of PCD boards and heating in the formic acid furnace at 250 °C for 60 s. The formic acid creates a reducing environment, preventing the oxidation of the Cu pads and molten solders during the soldering process.

### 2.3. Characterization

The interfacial structures and phases of the Sn-Cu-Bi-In alloys and solder joints were analyzed using scanning electron microscopy (SEM, Merlin Compact, Zeiss, Oberkochen, Germany) and transmission electron microscopy (TEM, Talos f200×, FEI, Waltham, MA, USA). FIB (ThermoFisher, V400ACE, Waltham, MA, USA) was used to fabricate the sample for TEM characterization. The tensile strength of the solder alloys was measured using an electronic universal testing machine (AG-X plus, Shimadzu, Kyoto, Japan). The parameters for testing mechanical properties of the solder joints are displayed in Table 2, referring to JESD22-B117B [30] and IEC 60068-2-6 [31] standards. The shear strength of the solder joint was tested using a push–pull tester (Nordson DAGE 4000, Aylesbury, UK) with a speed of 100 μm/s. The vibrational stability was tested via a vibration platform system (ES-6-230, Dongling, Suzhou, China). The mechanical properties were measured by testing 5 samples for each parameter.

## 3. Result and Discussion

### 3.1. The Characterization of Sn-Cu-Bi-In-Ni Solder Alloys

**Figure 2:**
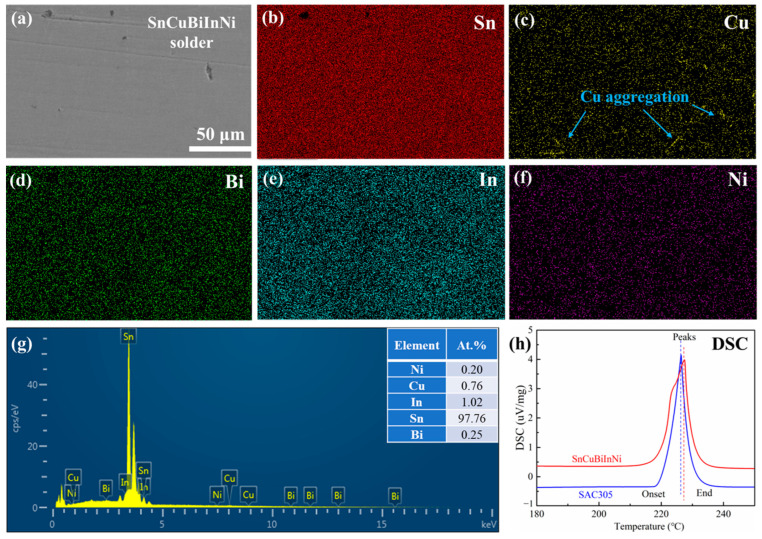
Characterization of as-prepared Sn-Cu-Bi-In-Ni solders: (**a**) SEM images, element map distribution of (**b**) Sn, (**c**) Cu, (**d**) Bi, (**e**) In, and (**f**) Ni; (**g**) EDS spectra of the whole region in (**a**); (**h**) DSC results of Sn-Cu-Bi-In-Ni and SAC305 solders.

The microstructures of the as-prepared Sn-Cu-Bi-In-Ni alloys are shown in Figure 2a, accompanied by the corresponding element map distributions in Figure 2b–f. Across the entire SEM image, the Sn element exhibit uniform distribution (shown in Figure 2b). A slight aggregation of Cu elements can be observed in Figure 2c, presumably representing a Cu-Sn compound. The element maps of Bi, In, and Ni are displayed in Figure 2d–f, respectively. The uniform distribution of these elements is advantageous for consistent solder performance. The EDS spectrum for the entire area depicted in Figure 2a is displayed in Figure 2g. Peaks corresponding to Sn, Cu, Bi, In, and Ni are evident, with atomic percentages of 97.76%, 0.76%, 0.25%, 1.02%, and 0.2%, respectively. The discrepancies between the EDS results and the alloy’s nominal chemical component can be primarily attributed to detection errors, particularly for elements with the percentages below 1%.

In practical applications, the melting point of solders play a crucial role. Figure 2h illustrates the DSC curves of the as-prepared Sn-Cu-Bi-In-Ni solder alloys, alongside the DSC results of SAC305 solders for comparative purposes. The initial melting temperature of SAC305 is 219.2 °C, and its ending point is 228.6 °C. For Sn-Cu-Bi-In-Ni solder alloys, the initial melting temperature is 216.5 °C with a thermal absorption peak at 227.4 °C, which is 1 °C higher than that of SAC305 solders. The addition of Ni may elevate the melting point, whereas In and Bi element tend to lower the melting point of Sn-based alloy [32,33], thereby maintaining the melting range of the prepared Sn-Cu-Bi-In-Ni solder alloys.

**Figure 3:**
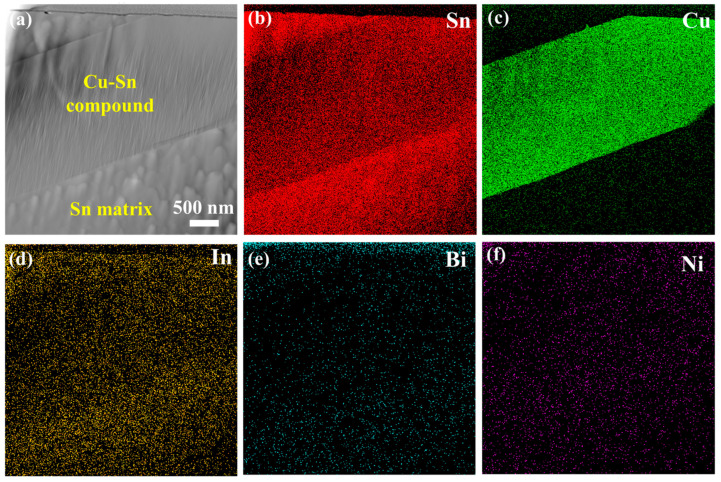
(**a**) STEM image of Sn-Cu-Bi-In-Ni solders, corresponding element map distribution of (**b**) Sn, (**c**) Cu, (**d**) In, (**e**) Bi, and (**f**) Ni.

To further investigate the composition and phases of the Sn-Cu-Bi-In-Ni alloys, the TEM analysis was conducted using FIB for sample preparation. Figure 3a presents the STEM image acquired from the Sn-Cu-Bi-In-Ni alloys, with corresponding element maps shown in Figure 3b–f. The distributions of Sn and Cu indicate the interface between the Sn matrix and Cu-Sn compound, and the In content is slightly higher in the Sn region due to its good solubility in Sn. The Ni and Bi elements are uniformly distributed through the region, suggesting no segregation in the Sn-Cu-Bi-In alloys.

**Figure 4:**
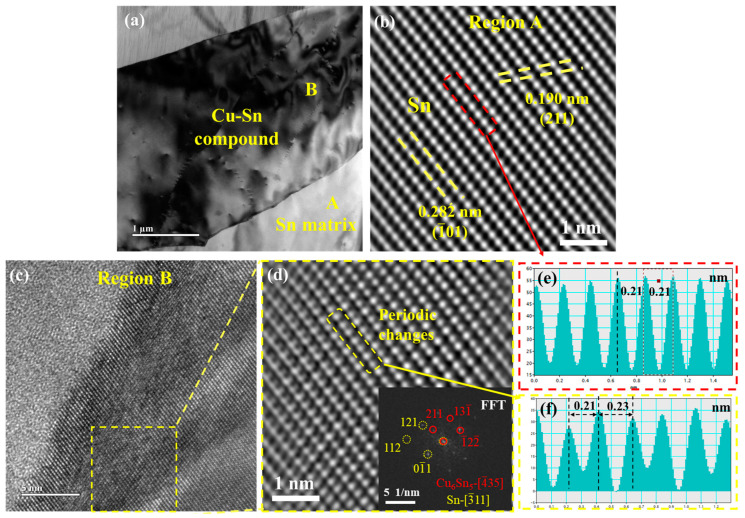
(**a**) Bright-field TEM image of Sn-Cu-Bi-In-Ni solder, HRTEM images of (**b**) region A and (**c**) region B in (**a**), (**d**) magnified image of selected region in (**c**) (the inset image is the FFT pattern of the selected region in (**c**), and the circles are for pointing the diffraction spots out), atomic spacing in the selected region of (**b**): (**e**,**d**): (**f**).

Figure 4a displays the bright-field TEM image of the Sn-Cu-Bi-In-Ni alloy. The Sn matrix is marked as region A, and Cu-Sn is marked as region B. Figure 4b shows the high-resolution transmission electron microscopy (HRTEM) image corresponding to region A, revealing a uniform atomic distribution. The (211) and ($\overset{-}{1}$01) planes of β-Sn can be identified, aligning with the Sn matrix of the Sn-Cu-Bi-In-Ni alloy. The HRTEM image of region B in Figure 4a is presented in Figure 4c. The selected region in Figure 4c undergoes Fast Fourier Transform (FFT) and Inverse Fast Fourier Transform (IFFT) processing, with the IFFT result shown in Figure 4d. The atomic arrangement in Figure 4d differs in Figure 4b. The atomic spacing in the selected regions of Figure 4b are displayed in Figure 4e and 4f, respectively. It is evident that the atomic arrangement in Figure 4d exhibits periodic changes. The inset image in Figure 4d shows the corresponding FFT result, indicating the presence of β-Sn and Cu_6_Sn_5_ with zone axes of [$\overset{-}{3}$11] and [$\overset{-}{4}$35], respectively. The (0$\overset{-}{1}$1) interplanar spacing of β-Sn is equivalent to the ($\overset{-}{1}$2$\overset{-}{2}$) spacing of Cu_6_Sn_5_. Therefore, these two phases can be identified as uniformly mixed on an atomic scale, forming near-eutectic structures.

**Figure 5:**
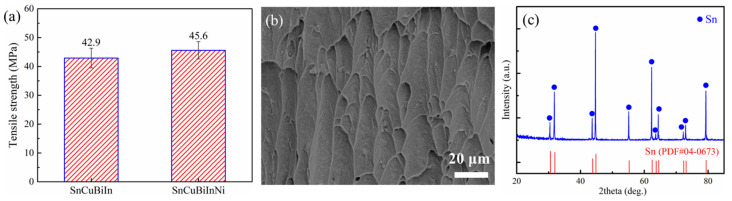
(**a**) Tensile strength of SAC305, Sn-Cu-Bi-In and Sn-Cu-Bi-In-Ni solders, (**b**) fracture morphology of Sn-Cu-Bi-In-Ni solders, (**c**) XRD patterns of the Sn-Cu-Bi-In-Ni solders.

To assess the mechanical properties of the as-prepared Sn-Cu-Bi-In-Ni solder alloy, tensile strength testing was conducted, with the results presented in Figure 5a. For comparison, the tensile strength of Sn-Cu-Bi-In solders, which was obtained from our previous research, is also included. The average tensile strengths of Sn-Cu-Bi-In and Sn-Cu-Bi-In-Ni are 42.9 MPa and 45.6 MPa, respectively. The incorporation of trace amounts of Ni could further bolster the strength by solution strengthening and grain size reduction, thereby enhancing the strength of Sn-based solder [25]. In addition, the morphologies and properties of the IMC could also be affected [34]. Figure 5b shows the fracture morphology of the Sn-Cu-Bi-In-Ni solders after the tensile test. The dimples indicate the good strength and plasticity of as-prepared solders. The XRD pattern (shown in Figure 5c) corresponds to the peaks of Sn, and no other peaks could be observed. The reason can be attributed the insufficient content of other elements, which is not enough to generate enough detection signal. However, the peaks exhibit a left shift compared to PDF#04-0673, resulting from the doping of other elements and their compounds. The electrical resistance of solders plays an important role in the electronic packaging. In principle, the addition of Bi or In element into Sn-Cu system could increase the electrical resistance. The Sn-0.7Cu-1Bi-3In exhibits the electrical resistance of 13.8 μΩ·cm [29]. After introducing 0.05 wt.% Ni, the resistance increases to 14.1 μΩ·cm, which is higher than before but still meets the demand.

### 3.2. Interfacial Structures and Phases of Sn-Cu-Bi-In-Ni Solder Joints on Cu Pads

**Figure 6:**
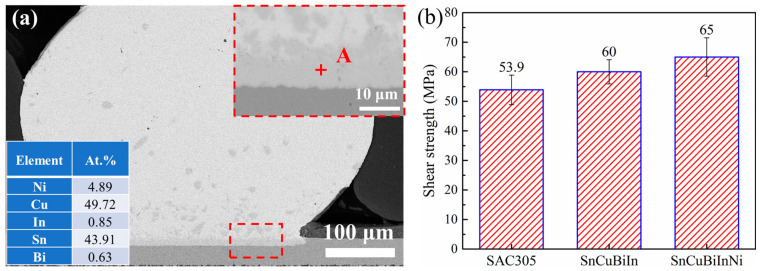
(**a**) Cross-sectional SEM image of the Sn-Cu-Bi-In-Ni solder joints on Cu pads (the inset table is the EDS result of Point A); (**b**) shear strength of the joints prepared by three kinds of solders.

Figure 6a shows the SEM of the joint prepared using Sn-Cu-Bi-In-Ni solders on Cu pads. The Sn ball is well bonded with the Cu substrate. A dark layer, identifiable as the IMC layer, is observed between Sn ball and Cu substrate. Within the Sn ball, several small clusters of compounds are distributed. The atomic percentages of Sn, Cu, and Ni at Point A are 43.91%, 49.72%, and 4.89%, respectively, suggesting the presence of Ni in the Cu_6_Sn_5_ compound, preliminarily indicated as (Cu,Ni)_6_Sn_5_. The mechanical properties of the solder joints are evaluated by shear testing and presented in Figure 6b, alongside the shear strength of SAC305 and Sn-Cu-Bi-In joints for comparison. The average shear strength of the as-prepared Sn-Cu-Bi-In-Ni solder joint is approximately 65 MPa, higher than the other two types of solders. The confidence interval (95%) can be calculated as 65 ± 5.7 MPa. Compared to reference [29], adding Ni element could obviously increase the shear strength of solder joints. These findings confirm that the incorporation of Ni enhances the mechanical strength of solder joints.

**Figure 7:**
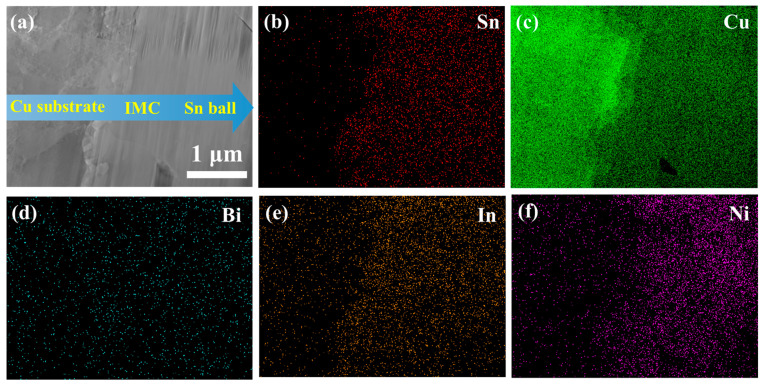
(**a**) STEM image of the Sn-Cu-Bi-In-Ni solder joint, corresponding element map distribution of (**b**) Sn, (**c**) Cu, (**d**) Bi, (**e**) In, and (**f**) Ni.

The TEM sample taken for the solder joint is achieved using FIB. Observing Figure 7a from left to right, three distinct regions are evident, with their corresponding element maps shown in Figure 7b–f. The Sn and Cu element maps indicate that these regions correspond to Cu pad, IMC layer, and Sn ball, respectively. Due to its low content, no obvious Bi aggregation is detectable. In and Ni elements are present in the region of IMC and Sn ball.

**Figure 8:**
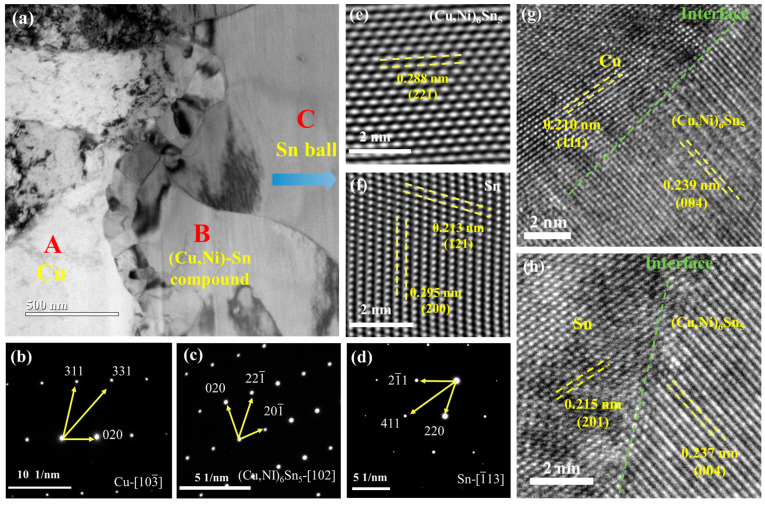
TEM analysis of the Sn-Cu-Bi-In-Ni solder joint. (**a**) Bright-field TEM image, SAED patterns of (**b**) Cu substrate, (**c**) (Cu,Ni)-Sn compound, and (**d**) Sn ball; HRTEM images of (**e**) (Cu,Ni)-Sn compound and (**f**) Sn ball; interfacial regions of (**g**) Cu/(Cu,Ni)_6_Sn_5_ and (**h**) Sn/(Cu,Ni)_6_Sn_5_.

Figure 8a illustrates the bright-field TEM image of the solder joint. The selected area electron diffraction (SAED) patterns taken from the three regions marked from A to C are shown in Figure 8b–d. The SAED pattern corresponding to region A can be indexed as Cu with the zone axis of [10$\overset{-}{3}$], as shown in Figure 8b. Figure 8c reveals that the SAED pattern of region B agrees with the lattice of Cu_6_Cu_5_. Combined the EDS results from Figure 6a and Figure 7, it is concluded that the IMC layer can be attributed to the (Cu,Ni)_6_Sn_5_ phase with the zone axis of [102], indicating that some Cu sites in the lattice have been substituted by Ni atoms. Shown in Figure 8d is the SAED pattern (region C) of the β-Sn with the zone axis of [$\overset{-}{1}$13], corresponding to the Sn ball in the joint. Figure 8e shows the HRTEM image of (Cu,Ni)_6_Sn_5_ IMC layer, agreeing with the (221) plane. The (200) and (121) planes of β-Sn are displayed in the HRTEM image of Figure 8f. Figure 8g illustrates the HRTEM image of the Cu/(Cu,Ni)_6_Sn_5_ interface, showing the Cu (111) and (Cu,Ni)_6_Sn_5_ (004) planes. The HRTEM image of the Sn/(Cu,Ni)_6_Sn_5_ interface is shown in Figure 8h, depicting the Sn (201) and (Cu,Ni)_6_Sn_5_ (004) planes. Additionally, the presence of In is detected in Figure 6a and Figure 7e, indicating the doping of In in Cu_6_Sn_5_, which could potentially enhance the IMC properties. Due to the limited content of In, Ni is the main doping element, and the compound is still written as (Cu,Ni)_6_Sn_5_.

**Figure 9:**
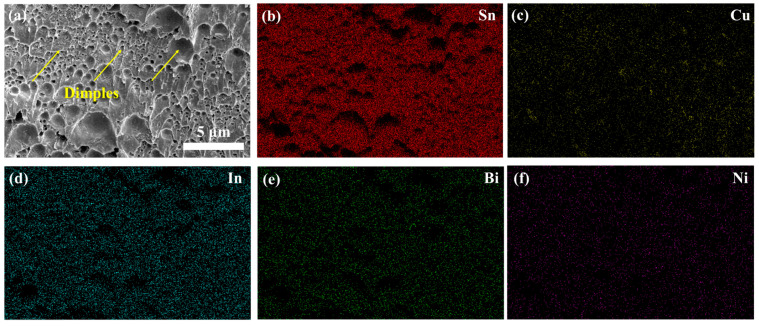
(**a**) SEM image of the fracture of Sn-Cu-Bi-In-Ni joints, corresponding element map distribution of (**b**) Sn, (**c**) Cu, (**d**) In, (**e**) Bi, and (**f**) Ni.

The fracture surface of the solder joint after shear test is characterized by SEM and EDS. Figure 9a shows the morphology of the fracture, revealing numerous dimples, which suggest good plasticity of the solder joint. Figure 9b–f display the corresponding element map distributions of Sn, Cu, In, Bi, and Ni elements, and no significant aggregation is observed.

### 3.3. Vibration Test of Solder Joints and the Mechanism of Reinforcement

**Figure 10:**
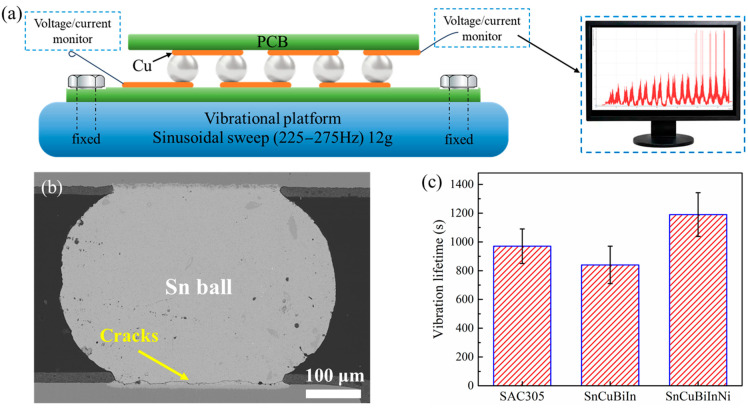
Vibration test of the Sn-Cu-Bi-In-Ni solder joints: (**a**) schematic diagram of vibration platform, (**b**) SEM image of the interfacial cracks after vibration test, (**c**) vibrational lifetime of different solder joints.

In practical applications of electronic products, it is necessary to ensure the stability of solder joints under dynamic loads. As shown in Figure 10a, the vibration test employs a sinusoidal sweep frequency acceleration ranging from 225 to 275 Hz, with a load of 12 g applied at room temperature. In the earlier experiment, a larger range of frequency was applied to test the vibrational stability. The results proved that the frequencies 225–275 Hz help to excite the resonance of PCB boards in the packaging, indicating that this range could effectively and efficiently evaluate the vibrational stability. The load was chosen by following the same method. The amplitude of the vibrations was 0.05 mm, as shown in Table 2. The PCB board, featuring a series of solder joints, is securely mounted on the vibration platform. These solder joints are connected in series. During the vibration test, an electric current flows through Cu pads and solder joints, while the voltage and current are continuously monitored. If a significant increase in resistance is observed, it indicates a failure in the solder joints. Notably, the sample appearance is not obviously changed after failure because only a part of solder joints fails, indicating that the chip and PCB board are still joined.

Figure 10b shows the cross-sectional interface of the solder joint that failed after undergoing a vibration test. At the bottom, cracks can be distinguished, potentially increasing the resistance of the packaging system. Figure 10c displays the vibrational lifetime of the as-prepared Sn-Cu-Bi-In-Ni solder joints. For comparison, SAC305 and Sn-Cu-Bi-In solder joints were also subjected to vibration testing under identical experimental conditions. The average lifetimes of SAC305 and Sn-Cu-Bi-In solder joints were approximately 970 s and 840 s, respectively. In the case of Sn-Cu-Bi-In solders, the Bi element enhances strength but also introduces brittleness, thereby compromising toughness and vibration stability [35]. By incorporating Ni into Sn-Cu-Bi-In, the vibration lifetime of the solder joints was extended to approximately 1200 s with the standard deviation of 152 s, higher than SAC305 and Sn-Cu-Bi-In solder joints. The confidence interval (95%) can be calculated as 1190 ± 133.2 s. The strengthening mechanism associated with Ni addition can be attributed to several factors.

The cracks resulting from vibration failure are indicative of fatigue fracture, which is significantly affected by residual stress [36,37]. The area with the highest residual stress typically resides at the corner of the Sn ball adjacent to the IMC layer, aligning with the fatigue crack initiation points shown in Figure 10b. Consequently, the mechanical properties of the Sn ball and the distribution of residual stress emerge as the two primary factors. The incorporation of Ni element, uniformly distributed in the solder alloy, provides a large amount of nucleation number during solidification, thereby refining the grain structure. The refined grains exhibit both good strength and toughness, enhancing the shear strength and vibration stability of the solder joints. Moreover, the dissolution of Ni contributes to solid solution strengthening within the Sn ball.

The residual stress in the Sn-Cu-Bi-In-Ni solder joint results from the thermal mismatch among Cu pads, IMC layer, and Sn ball. The phase diagram of five elements is difficult to achieve, but it can be analyzed based on the current Sn-Cu-Ni phase diagram [38]. In this work, the IMC layer has been proven to be (Cu,Ni)_6_Sn_5_ instead of Cu_6_Sn_5_ after adding Ni to the solders. Cu_6_Sn_5_ exists in the monoclinic η’-Cu_6_Sn_5_ and hexagonal η-Cu_6_Sn_5_, corresponding to the lower-temperature phase and higher-temperature phase, with a phase transition temperature of 186 °C [39]. In our study, the (Cu,Ni)_6_Sn_5_ still shows the monoclinic phase at room temperature. However, doping the Ni in Cu_6_Sn_5_ can still stabilize the phase to some extent, thereby potentially reducing the thermal expansion of the IMC layer [40]. Consequently, this stabilization can alleviate residual stress in the joint, enhance vibrational stability, and improve the shear strength of the solder joints. Moreover, the (Cu,Ni)_6_Sn_5_ phase also exhibits better mechanical properties than Cu_6_Sn_5_ [41]. The nanoscale grain size of the IMC layer depicted in Figure 8a contributes positively to its mechanical properties. The interface between the IMC layer and the Sn ball often serves as the area where cracks propagate during vibration test. Notably, the (Cu,Ni)_6_Sn_5_ is tightly bonded with Sn ball shown in Figure 8h, which is conducive to the stability of the IMC/Sn interface.

## 4. Conclusions

In summary, we prepared the Sn-Cu-Bi-In-Ni solder, analyzed the phases and mechanical properties, characterized the interfacial microstructures and mechanical stability of the solder joints. The Sn matrix and Cu_6_Sn_5_ compound were found to be mixed at the atomic scale in the as-prepared solder alloys. Additionally, the Ni element was uniformly distributed throughout the solder alloy. The Sn-Cu-Bi-In-Ni alloy exhibited an average tensile strength of 45.6 MPa, slightly surpassing that of Sn-Cu-Bi-In. After reflow soldering, the Sn-Cu-Bi-In-Ni solder joints demonstrated an average shear strength of 65 MPa. Notably, the vibration lifetime increased from 840s to 1200s upon the addition of Ni to Sn-Cu-Bi-In. The enhancement in mechanical properties of the Sn-Cu-Bi-In-Ni solder joints due to Ni can be attributed to solution strengthening, grain refinement, and the formation of (Cu,Ni)_6_Sn_5_ IMC layer. Compared to Cu_6_Sn_5_, (Cu,Ni)_6_Sn_5_ exhibits superior mechanical properties and lower thermal expansion, which helps to reduce residual stress, improve shear strength, and enhance vibration stability. More research, such as the effect of multi-field coupling on the solder joints of Sn-Cu-Bi-In-Ni, can be carried out in future work. The theoretical calculation of the synergies of multi-elements is also a challenging and meaningful research direction.

## Figures and Tables

**Table 1:** Chemical component of the Sn-Cu-Bi-In-Ni solders.

Element	Sn	Cu	Bi	In	Ni
Wt.%	Bal.	0.7%	1%	3%	0.05%

**Table 2:** Parameters of the mechanical tests for the Sn-Cu-Bi-In-Ni solder joints.

Shear Test	Speed	Vibration Test	Frequency	Load	Amplitude
Solder joints	100 μm/s	Chips	225–275 Hz	12 g	0.05 mm

## Data Availability

The original contributions presented in this study are included in the article. Further inquiries can be directed to the corresponding authors.
